# Protein Complex Evolution Does Not Involve Extensive Network Rewiring

**DOI:** 10.1371/journal.pcbi.1000132

**Published:** 2008-07-25

**Authors:** Teunis J. P. van Dam, Berend Snel

**Affiliations:** 1 Theoretical Biology and Bioinformatics Group, Department of Biology, Utrecht University, Utrecht, The Netherlands; 2 Department of Physiological Chemistry and Center for Biomedical Genetics, University Medical Center Utrecht, Utrecht, The Netherlands; 3 Academic Biomedical Centre, Utrecht University, Utrecht, The Netherlands; Weizmann Institute of Science, Israel

## Abstract

The formation of proteins into stable protein complexes plays a fundamental role in the operation of the cell. The study of the degree of evolutionary conservation of protein complexes between species and the evolution of protein-protein interactions has been hampered by lack of comprehensive coverage of the high-throughput (HTP) technologies that measure the interactome. We show that new high-throughput datasets on protein co-purification in yeast have a substantially lower false negative rate than previous datasets when compared to known complexes. These datasets are therefore more suitable to estimate the conservation of protein complex membership than hitherto possible. We perform comparative genomics between curated protein complexes from human and the HTP data in *Saccharomyces cerevisiae* to study the evolution of co-complex memberships. This analysis revealed that out of the 5,960 protein pairs that are part of the same complex in human, 2,216 are absent because both proteins lack an ortholog in *S. cerevisiae*, while for 1,828 the co-complex membership is disrupted because one of the two proteins lacks an ortholog. For the remaining 1,916 protein pairs, only 10% were never co-purified in the large-scale experiments. This implies a conservation level of co-complex membership of 90% when the genes coding for the protein pairs that participate in the same protein complex are also conserved. We conclude that the evolutionary dynamics of protein complexes are, by and large, not the result of network rewiring (i.e. acquisition or loss of co-complex memberships), but mainly due to genomic acquisition or loss of genes coding for subunits. We thus reveal evidence for the tight interrelation of genomic and network evolution.

## Author Summary

Protein complexes are a pivotal part of the functioning of cells in health and disease. Studying the evolution of these essential cellular features is of great intrinsic as well as practical interest. However, the study of the evolution of protein complexes by comparative analysis is fraught with difficulties. Hence current reports that reveal low overlap in the interactome between species are often reluctant to equate this low level of overlap to a low level of conservation. Here we exploit new public data sets, which display unparalleled coverage, to study the amount of co-complex membership conservation, and we present a novel measure for the absence of interactions. We thereby observe a hitherto unreported high level of conservation of 90% of the interactions when the presence of the genes coding for the protein pairs that participate in the same protein complex is also conserved. This allows for new insights into the evolution of protein complexes: the evolutionary dynamics of protein complexes are, by and large, not the result of network rewiring (i.e. acquisition or loss of co-complex memberships), but mainly due to genomic acquisition or loss of genes coding for subunits.

## Introduction

Many proteins perform their functions together with other proteins to form distinct complexes which are responsible for specific processes in a cell. Understanding how, why and when proteins associate into stable protein complexes is a pivotal part of understanding cellular life. The evolution of protein complexes is intrinsically of interest, as protein complexes are important functional units. In addition, evolutionary information can help us to clean noisy high-throughput data on protein complexes and interactions [1],[2]. In general, measuring the evolutionary dynamics of protein complexes should improve the framework for function prediction and comparative analysis of interactome networks. For example, knowledge on interactome evolution can help us to establish how reliably we can transfer measured interactions of a protein in *S. cerevisiae* to its ortholog in Human for function prediction.

Various aspects of the evolution of protein complexes and interactomes have been studied [3]. Work on interaction networks so far has revealed that highly connected proteins tend to be more conserved than less connected proteins when looking for the presence or absence in other species [4]. Also, higher connected proteins tend to evolve slower than less connected proteins [5]. Moreover it has been shown that the subunits of protein complexes seem to evolve uncohesively: the genomes of many species contain only a subset of the genes that make up a protein complex of a particular species [6],[7]. However, all these studies did not analyze the evolution of interactions or co-complex membership, but only the evolution of the genes.

The actual conservation of protein interactions themselves is still debated, in part because information and direct measurements of interactions in multiple species is sparse. Suthram and co-workers [8] for instance, have found remarkably low overlap in interaction networks between *P. falciparum* and other eukaryotic interaction networks, like those of yeast and human. They also concluded that even between closer and well studied eukaryotes like *S. cerevisiae* and *D. melanogaster*, many interactions and complexes have been lost. This study, and others like it, has been careful to equate small overlap with a low degree of conservation and has pointed out that the analysis of complex evolution has been hampered by the quality of the available high throughput data. In contrast anecdotal evidence based on specific cases studied from the literature suggest high conservation of co-complex membership such as observed in the ribosome [9]. Therefore it remains unresolved to what extent protein interactions and protein complexes are conserved.

When analyzing interaction conservation we need to acknowledge that proteins can keep, lose or gain interactions. To properly measure interaction conservation we need data which not only contains protein-protein interactions but also contains data on proteins which do not seem to interact [6]. The measurements as done in interaction experiments initially provided data on the former. Yet when the coverage of the data is such that it approximates ‘complete’, the probability that a protein pair without measurable interaction does indeed not interact should increase rapidly.

With the publication of two new datasets of high throughput tandem affinity purification-mass spectrometry (TAP-MS) experiments in *S. cerevisiae* [10],[11], data has become available which is seemingly of high enough quality [11],[12] to warrant a new look at interaction conservation. We revisit therefore the question of how complexes evolve and how well protein-protein interactions are conserved.

Measuring evolution of protein complexes obviously depends on a reasonable definition of what constitutes a complex: proteins can associate strongly to other proteins and form a stable protein complex (e.g. proteasomes) or proteins can associate transiently to often many other proteins (e.g. a kinase and its substrate) and not be truly part of one stable complex. We chose to study the evolution of the first (stable) type. In addition new insights propose a world view where complexes are not static entities but fluctuate in time and space [10]. Unlike the manner in which it is by necessity stored in reference databases such as MIPS or SGD, the composition of protein complexes is condition and sub cellular localization dependent. This also makes it difficult to study the evolution of protein complexes; i.e. if only a subset of the subunits is involved in a complex in another species, is the complex then conserved? We here adapt to the latter problem by choosing as the unit of which we want to measure conservation “a pair of proteins that are part of the same protein complex”. For brevity we will refer to this as “co-complex membership” or sometimes the even shorter and arguably inappropriate term “interaction”.

In this study we extend interaction data by defining non-interactions in order to examine co-complex membership conservation between *S. cerevisiae* and Human. Estimating the absence of interactions allows us to look at the conservation and not just the overlap between two interaction networks. The analysis reveals that the main processes of evolution for complexes are the acquisition of new or the loss of old subunits as the co-complex interaction network is highly conserved between orthologous proteins in *S. cerevisiae* and Human.

## Results

### Dataset Quality and False Negative Rate Assessed by Yeast Complexes

The new TAP-MS datasets seem to be very complete and accurate [10]–[12]. We explicitly test the completeness of the datasets by specifically analyzing to what extent different HTP datasets are able to predict all interactions and absence of interactions, i.e. the false negative rate (type 2 error). A false negative will result in the observation that an association is absent while in reality the interaction is present but the experiment failed to detect it. We use the false negative rate because it is a measure of how complete the actual connectivity of a given protein is represented in the datasets. Such false negative pairs are crucial for the study of evolution, because these false negatives will erroneously lower the degree of conservation.

A reference set of known complexes is needed to assess which co-complex memberships are erroneously reported as absent in the various HTP datasets (false negatives). In the light of the ongoing discussion on what constitutes a complex [10],[11], we used different independent sources such as MIPS and SGD and their intersection (see Table 1). We use the latter as the main reference, because it provides a reference set in which both MIPS and SGD agree and therefore more is reliable in terms of co-complex memberships and complex definition.

**Table 1:** Overview of Complex Definitions.

Definition	Reactome	MIPS	SGD GO
Source	Reactome Database	MIPS Database	SGD Database
Processing	“direct complex” interactions	Subunits pooled by complex ID	By GO category
Date	9/19/2006	5/18/2006	5/9/2007
Nr of Complexes	391	217	225
Min Complex size	2	2	2
Max Complex size	140	81	94
Avg. Complex size	7.72	6.33	7.55
Median	2	4	4
Co-complex memberships	5960	15613	19073
Proteins	973	1194	1467

Naturally, there is a trade-off between the false negative rate and false positive rate when choosing an appropriate cut-off value for the TAP-MS datasets. The optimal cut-off value for the socio-affinity scores was determined by plotting a Receiver-Operator Curve (see Text S1). We found that a relatively low cut-off value of 0 provides an optimal balance between specificity and sensitivity for measuring complex interactions.

**Figure 1:**
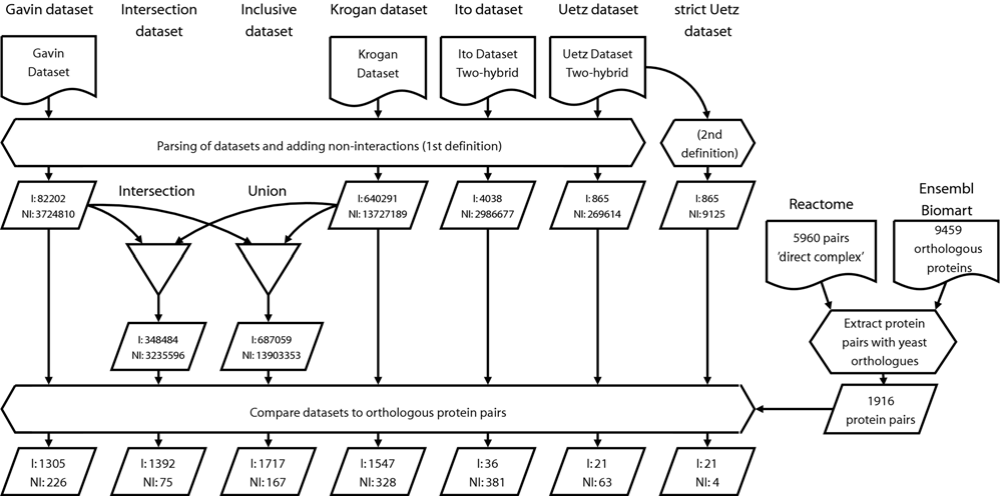
Data flow diagram. NI = non-interaction, I = interaction. The non-interactions are calculated for each dataset before they are combined in a union or intersection dataset. The complex definition of Reactome and ortholog definitions from Ensembl are combined to find the conserved protein pairs. The interaction data of the conserved protein pairs are extracted from the datasets and the interaction conservation is calculated.

We observe that the new datasets achieve very low false negative rates. The Gavin dataset has a false negative rate of 0.23 whereas the Krogan dataset has a false negative rate of 0.32 (Table 2). Combining the TAP-MS datasets (both union and intersection) does not only increase the number of true positives but also reduces the number of false negatives and consequently the false negative rate (Table 2), e.g. the intersection of the Gavin and Krogan datasets has a false negative rate of 0.11 (see Figure 1 and Materials and Methods on dataset construction). These low false negative rates reveal that when the TAP-MS datasets report an absence of interaction only a small percentage is a “failure” of the experimental assay. The new datasets are therefore a substantial improvement for the study of co-complex membership conservation relative to what was available previously.

**Table 2:** False Negative Rates for Different Datasets Compared to Complex Definitions.

*Datasets*	*Intersection of MIPS and GO*			*MIPS*			*SGD GO*		
	FNR*	*#FN* ^¶^	*#TP* ^¥^	FNR*	*#FN* ^¶^	*#TP* ^¥^	FNR*	*#FN* ^¶^	*#TP* ^¥^
Gavin et al.	0.23	1226	4083	0.33	2284	4687	0.37	3769	6328
Krogan et al.	0.32	2209	4644	0.44	4208	5372	0.52	7927	7406
Intersection	0.11	517	4396	0.21	1356	5203	0.25	2378	7233
Inclusive	0.21	1517	5732	0.34	3370	6622	0.42	6572	9247
Uetz et al.	0.66	91	46	0.75	194	63	0.76	270	87
Uetz et al. strict	0.1	5	46	0.11	8	63	0.15	15	87
Ito et al.	0.92	822	76	0.93	1427	114	0.95	2358	114

*False Negative Rate, ¶False Negatives, ¥True Positives.

In addition to the TAP-MS datasets we also analyzed other high-throughput Yeast-2-Hybrid datasets (Y2H) by Uetz et al. [13] and Ito et al. [14] in order to compare them to the new datasets (for an overview on all datasets see Table 3). We see that the false negative rate in these Y2H assays is much higher, when we define absence of an interaction from Y2H conventionally: that is to say an absence is a prey and bait pair that failed to report an interaction. The higher false negative rate of the Y2H datasets is of course to be expected because Y2H measures **direct** protein-protein interactions rather than co-complex memberships. Mass-spec co-purifications are expected to retrieve co-memberships more easily [15]. At the same time it might also be that Y2H does have a slightly higher natural level of false negatives as implied previously [2]. To test this, we redefined our Y2H negatives for the Uetz dataset as follows: both the bait-prey as the prey-bait has been tested and both failed to report an interaction. We see a very dramatic decrease in the false negative rate for the Uetz ‘strict’ dataset (Table 2). In fact Uetz strict has a false negative rate comparable to the intersection of the two mass-spec datasets (0.10 for Uetz strict as compared to 0.11 for the Intersection of the Gavin and Krogan datasets, see Table 2). This shows it is possible to obtain reliable indications of the absence of an interaction from apparently less complete datasets. However, this requires specific attention to the method by which an absence of interaction is inferred from the primary data. Due to coverage of this Uetz strict dataset we cannot use it as the main source for the study of the conservation of interaction, but we can use it to test how general our findings from the mass-spec source are, and whether or not they depend on the precise experimental method for detecting interactions.

**Table 3:** Overview of PPI Datasets.

Datasets	Interactions	Non-interactions	Species	Source	Method	Advantages	Disadvantages
Gavin et al.	82202	3724810	Yeast	Gavin et al.	TAP-MS	Large datasets. Repeated purifications.	Does not detect low affinity interactions. Does not detect 1-to-1 interactions but clusters of proteins.
Krogan et al.	640291	13727189	Yeast	Krogan et al.	TAP-MS	′′	′′
Intersection	348484	3235596	Yeast	This publication	TAP-MS	′′	′′
Inclusive	687059	13903353	Yeast	This publication	TAP-MS	′′	′′
Uetz et al.	865	269614	Yeast	BioGRID	Y2H	Can also detect low affinity interactions. Measures 1-to-1 interactions.	Low coverage.
Uetz et al. strict	865	9125	Yeast	This publication	Y2H	′′	′′
Ito et al.	4038	2986677	Yeast	BioGRID	Y2H	′′	′′
Rual et al.	1911	614341	Human	IntAct	Y2H	′′	′′
Stelzl et al.	1967	249857	Human	IntAct	Y2H	′′	′′
Ewing et al.	5761	1804013	Human	IntAct	PI-HTMS	Larger than human Y2H datasets.	Purifications done only once.

### Interaction Network Evolution in Complexes

The Gavin and Krogan datasets and in particular the combination of these datasets (union and intersection) show a very low false negative rate: i.e. only a small fraction of the true co-complex memberships are not reported by these datasets. Given that these datasets are available with substantially improved false negative rates we have an excellent starting point for comparative genomics to see to what extent co-complex membership is conserved between species. Reactome for Human [16] was used as a highly reliable reference set for calculating interaction conservation. Reactome is a high quality manually curated database based on expert opinion. Recently a Co-IP interaction dataset has been published for the Human interactome by Ewing et al. [17]. We use this dataset as complementary source to confirm our qualitative trends, rather than our main reference set, because this dataset is only slightly larger than Reactome (6,463 interactions vs. 5,960), but has less protein pairs with orthologs in yeast (650 vs. 1,916) and contains experimental noise (see Text S1 for analysis performed with the Ewing dataset).

We extracted protein pairs that were part of the same core protein complex according to Reactome. Orthology data was extracted from Ensembl (see Materials and Methods) in order to transfer the yeast interaction data onto Reactome (Figure 1). This analysis revealed that out of the 5960 human co-complex memberships 4044 are absent in yeast due to the absence of either one (1,828) or both (22,6) of the interaction partners, leaving 1916 pairs with orthologs in yeast. In terms of complexes we found that 66% of human complexes contain less than 50% subunits with orthologs in yeast with an average of 35% over all complexes, which is similar to the percentage of protein pairs. These results are confirmed by orthology calculated with inparanoid [18] (see Text S1). Thus a large number of co-complex membership pairs are not conserved because either one or both of the genes was lost in fungi or acquired in animals. This is consistent with previous findings on the evolutionary cohesiveness of protein complexes [6]. Therefore a tremendous amount of flexibility in the evolution of protein complexes is not due to the evolution of the co-complex membership (the interactions) itself, but rather due to the acquisition and loss of subunits from the genome.

We subsequently asked how many of the 1,916 gene pairs are also part of the same protein complex in yeast and, more importantly, we also counted how many pairs are *not* interacting according to our inferred non-interacting pairs. In case of inparalogs conservation of interaction was inferred when one of the inparalogs returned a positive interaction from the datasets (see Materials and Methods). We observe a high rate of co-complex membership conservation: 82.5% to 85.2% for the Gavin and Krogan datasets respectively and 91.1% to 94.9% for the Inclusive and Intersection datasets respectively (Table 4). Although this seems in contrast to the Y2H datasets (Uetz dataset reaches 24.1%, Ito dataset 8.6%), the Uetz strict dataset returns 84% conservation. The Y2H thus in fact confirms the observation on conservation from the TAP-MS datasets.

**Table 4:** Conservation of Protein-Protein Interactions Defined by Reactome in Yeast.

*Datasets*	*Interactions*	*Non-interactions*	*Conservation* 1	*Coverage* 2	*Complex coverage by dataset* 3
Gavin et al.	1305	226	85.2%	68.1%	135
Krogan et al.	1547	328	82.5%	80.7%	150
Intersection	1392	75	94.9%	72.7%	133
Inclusive	1717	167	91.1%	89.6%	152
Uetz et al.	21	63	24.1%	1.1%	26
Uetz et al. strict	21	4	84.0%	1.1%	17
Ito et al.	36	381	8.6%	1.9%	65

*Human Datasets*	*Interactions*	*Non-interactions*	*Overlap* 1	*Coverage* 2	*Complex coverage by dataset* 3
Ewing et al.	16	434	3.6%	0.8%	56
Rual et al.	3	5	37.5%	0.2%	5
Stelzl et al.	4	79	4.8%	0.2%	15

1. Conservation and overlap is calculated as 100%^*^#Interactions/(#Interactions+#Non-interactions).

2. Coverage is calculated as 100%^*^#Interactions/1916.

3. Number of Reactome complexes which contribute to co-complex memberships with yeast orthologs.

The rate of conservation that we obtain from the protein purification experiment datasets are not based on a small subset of protein pairs but on a very large proportion of all associated protein pairs. The TAP-MS datasets have coverage of up to 90% when combined as the union of both datasets. The coverage of Reactome by the Krogan and Gavin datasets is substantial (81% and 68% resp.), whereas the Y2H datasets cover at most 2% (Ito dataset) of the 1916 orthologous protein pairs in Reactome. Moreover the conservation rates are based for e.g. the intersection on 133 distinct complexes (Table 4). From the high conservation rates as well as the percentage of coverage as determined from our analysis based on the TAP-MS datasets, we conclude that the evolution of protein complexes is mainly due to the acquisition or loss of subunits and not due to network rewiring.

Analogous to the yeast datasets and the yeast complex definitions, we analyzed the overlap of the human Co-IP [17] dataset and Y2H datasets [19],[20] with Reactome. To prevent bias we only took those Reactome gene pairs that have orthologs in yeast. The overlap between the human datasets and Reactome is surprisingly so small, that they perform worse than the Y2H datasets from yeast. The small coverage of the human datasets is perhaps caused by the fact that the human HTP interaction studies targeted proteins that are presumably of more interest to mammalian systems.

### Loss and Acquisition of Co-complex Associations in Human

From the high conservation rates as determined from our analysis we conclude that the evolution of protein complexes is mainly due to the acquisition or loss of subunits and not due to network rewiring. The non-conserved interactions are those associations between protein pairs that are present in yeast and human as orthologs but whose interaction seems to have been either lost in yeast or acquired in human.

These associations are potentially interesting because they tell us about the evolution of new interactions. Out of the 1884 associations covered by the inclusive dataset only 167 seem to be not conserved (see Table 4). We scanned this list manually searching for possible errors in annotation, false negatives and true negatives (actual non-conserved protein-protein interactions). Of the 167 protein pairs 139 pairs are present in the same complex in yeast according to GO and/or MIPS or based on literature. In other words, a large portion of these pairs seem to be a member of the same protein complex in yeast and human according to the literature, but were never co-purified in either Krogan or Gavin. I.e. these 139 are possible false negatives of the experimental assays rather than non-conserved interaction pairs. The remaining 28 non-conserved interactions (see Text S1) consist of errors in orthology of one gene (5 interactions), incorrect assignment of two proteins to a complex in Reactome (10 interactions) and possible neo-functionalisation after duplication in human (3 proteins, 13 interactions).

Based on the analysis of the proteins pairs which did not have an interaction according to the HTP datasets, it seems that the actual conservation of co-complex membership might be higher than follows from our analysis, because we mostly ran into potential errors in orthology assignment, conceptual issues in the curated database of Reactome, or false negatives in the HTP assay. Interestingly, in this analysis the three proteins which represent potentially new complex memberships, are all proteins which have retained the same or similar function as their orthologs in yeast but have acquired additional functions and interactions in human.

## Discussion

We have shown that with the publication of the TAP-MS datasets by Gavin et al. [10]and Krogan et al. [11] we now have datasets which are sufficiently large to reliably estimate the level of co-complex membership conservation. Specifically, we have shown that the false negative rate of these datasets can be reduced to 7%. This means that we are now able to do comparative network studies with substantially less coverage problems for the yeast interactome than previous studies. This is important as estimates of the level of co-complex membership conservation do not only depend on reliable measures for the presence of a link but also on reliable measures for the absence of a link.

Unfortunately similar interaction data is not available for other species. We have therefore chosen to use a curated interaction database called Reactome and extracted complex definitions. Combining the human Reactome complex definition and the interaction data for yeast reveals that the complex protein pairs which have been conserved in both species do not lose their interaction in contrast to what has been previously suggested [8],[21]. We conclude therefore that evolution of protein complexes does not involve extensive network rewiring, but is mostly due to loss of subunits and the acquisition of novel proteins.

**Figure 2:**
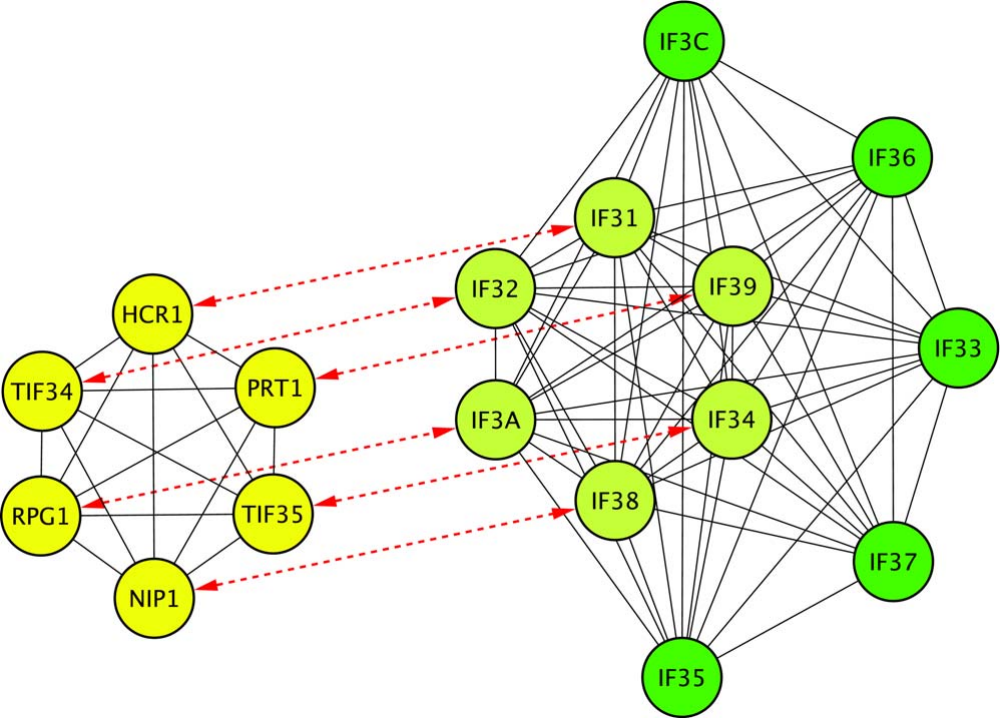
The eIF3 protein complex. The eIF3 complexes in yeast (yellow) and human (green) are depicted in a network with similar topography relevant to the orthologs (connected by red dotted lines). Although the eIF3 complex in human has expanded compared to yeast, all yeast proteins still have orthologs in the human eIF3 complex. Modification of the complex seems to have been mainly through the acquisition of new proteins.

This type of behavior is clearly illustrated by the eIF3 protein complex from human and its comparison to the complex in yeast (see Figure 2). The eIF3 complex in yeast (yellow) and human (green) are depicted in a network with similar topology relevant to the orthologs (connected by red dotted lines). Although the eIF3 complex in human has expanded compared to yeast, all yeast proteins are also part of the same complex in human (light green). Modifications of the complex during evolution have been through the acquisition of new proteins (green).

The high degree of co-complex membership conservation could potentially arise from some degree of circularity: the protein complexes in human have been originally identified in yeast. However, our knowledge of human complexes is not limited by what we know about complexes in yeast, as can be deduced by many human subunits which do not have orthologs in yeast such as *EF3C* or *IF36* in the example of the eIF3 protein complex (Figure 2). In general many human interactions are disrupted in yeast due to the absence of either one (1,828) or both (2,216) of the interaction partners. All these subunits are part of a complex in human but are absent in yeast. The knowledge about these subunits is the result of direct intensive biochemical analysis in human or other animal systems. Therefore, we have a substantial degree of trust in our estimate of interaction conservation, because the knowledge on the protein complexes deposited in Reactome is the result of direct extensive experimentation in animal systems and is not only based on experimentation in yeast.

An important aspect of protein-protein interaction evolution is that the physical interaction surface is often provided by distinct protein domains. In evolution of protein-protein interactions they play an important role as acquisition or loss of a particular domain can result in the combination of new interactions with new functions. Itzhaki et al. [22] report that 9% of protein-protein interactions in yeast and 20% in human can be ascribed to domain-domain interactions. It therefore bears to mind that a small part of co-complex membership conservation might not be due to the conservation of whole proteins but due to specific domains which have maintained the interaction. This would leave a conserved interaction network the freedom to add or change function without having to compromise interaction integrity. Another possible theoretical framework for our observations is given by Kirschner and Gerhart [23], who argue that conserved mechanisms or processes are conserved because they “deconstrain” phenotypic variations in other processes. Our observations neatly fit their theory: the conserved proteins and their conserved interactions represent a “backbone” to which variable subunits are observed to be added or removed.

The possible new interactions that we have found, *XAB2*, *PCBP1* and *PABP2*, still have the same or similar function as their yeast orthologs, but have acquired new functions and new interactions in human. Additions to the functionality were made only through minor instead of radical adjustments leaving the interaction network intact and added upon. In the light of co-complex membership this might imply that it is easier to add function and interactions than it is to remove the interaction while retaining the gene. The high conservation of co-complex memberships is also support for bioinformatic function prediction by transfer of information on complex-membership between orthologs: this aspect of gene function can be reliably transferred between evolutionary divergent species such as yeast and human when the partner gene is also present.

We have shown that the gain of interactions by existing proteins in complexes seems quantitatively not important in evolution. Rather the evolution of protein complexes is dominated by co-complex memberships that are acquired or lost concomitantly with acquiring or losing the gene. However, the precise order of events in the latter case is difficult to determine. If we for example suppose that the absence of an ortholog in yeast of a human protein complex member is the result of a gene loss (deletion) in the fungal lineage (rather than being acquired in animals), then there are two scenarios than can explain this loss. On the one hand the loss of membership to a protein complex could have preceded the evolutionary loss of the gene. On the other hand a co-complex membership is by definition disrupted by the deletion of the gene coding for the subunit from the genome.

For both examples divergence in transcriptional regulation could mediate less dramatic scenarios of interaction loss. Transcriptional regulation diverges significantly between relatively close species [24] and is therefore a faster process than for example gene loss or acquisition. Loss of membership could have preceded a fast transcriptional down regulation to avoid expression of potentially rogue proteins before the actual loss of the gene. If a subunit is no longer needed deletion of this subunit could have been preceded by down regulation, which could have given the organism some time to adapt (stabilize the complex) to the missing of the subunit before its deletion from the genome.

Although gene loss preceded by interaction loss seems somewhat more likely, the high level of co-complex membership conservation that we observe in those cases were the protein pairs are present in both species, suggest a low frequency of such evolutionary intermediate stages. Because we find such low frequency of intermediate stages and a high conservation rate of interactions between conserved proteins we reveal evidence of the tight interrelation of genomic and network evolution.

## Materials and Methods

### Interaction Datasets

#### Mass spec datasets

The Gavin dataset with socio-affinity scores was obtained from the embl website (http://yeast-complexes.embl.de/) as referred to in the original article [10]. The Krogan dataset has been obtained from Vera van Noort who kindly provided us with a processed tab delimited file in which the raw Krogan data had been converted into socio-affinity scores as defined by Gavin et al. [10]. For an overview of all interaction datasets used in this publication see Table 3.

#### Yeast-2-hybrid datasets *S. cerevisiae*

Yeast-2-Hybrid interaction data for *S. cerevisiae* was downloaded from BIOGRID (http://www.thebiogrid.org/ 01/03/2007). The Y2H datasets from Uetz et al. [13] and Ito et al. [14] were extracted by pubmed id.

#### Yeast-2-hybrid datasets for human

Stelz [20] and Rual [19] datasets were obtained from the IntAct database (http://www.ebi.ac.uk/intact/) on 01/12/2007. Files were downloaded in PSI MI 2.5 XML format and data was extracted by using XMLMakerFlattener.

#### Co-IP dataset for human

The Ewing [17] dataset was downloaded from the IntAct database on 01/14/2008. The four PSI MI XML files where parsed for primary UNIPROT identifiers. Id's of proteins which did not have a primary identifier where retrieved from the UNIPROT database by blast (100% identity, lowest E-value).

#### Defining non-interactions

We have defined absence as interactions between proteins that have been successfully purified as either bait or prey, but have not occurred together. For the ‘Uetz strict’ dataset we have defined absence of interactions between proteins that have been successfully purified as both bait and prey, but have not occurred together. ‘Absence of interaction’ was included into the datasets by assigning the protein pair the socio-affinity score 0 which did not occur in each of the original datasets.

#### Combining the mass-spec datasets

The Gavin and Krogan datasets were combined in two ways. Firstly the Intersection dataset represents the intersection of protein pairs of both Gavin and Krogan datasets after the addition of non-interactions. The socio-affinity scores were averaged. Secondly the Inclusive dataset represents the union of protein pairs of both Gavin and Krogan datasets after the addition of non-interactions. The socio-affinity scores where averaged where appropriate. It may be noted that the total number of positive interactions in the intersection dataset is larger than the Gavin dataset. This is because the dataset was combined by identical protein pairs which allowed for many interactions in Krogan to be included which are non-interactions in Gavin.

### Complex Definitions

#### Yeast complex definitions

For an overview of all complex definitions in this publication, see Table 1.

The MIPS complex definition was downloaded from ftp://ftpmips.gsf.de/yeast/catalogues/complexcat, last updated 05/18/2006. Proteins were pooled per complex ID and interactions were defined between proteins which are present in the same complex.

The SGD Gene Onthology (GO) complex definition was provided by Patrick van Kemmeren. SGD GO (as of 05/09/2007) was parsed, keeping only those components which contain the following words in their GO description: complex, subunit, ribosome, proteasome, nucleosome, repairosome, degradosome, apoptosome, replisome, holoenzyme or snRNP. Only the lowest annotation level was maintained. Associations that were obtained from high-throughput data have been removed to avoid pollution with false positive interactions. Specifically the following publications were excluded: Ito et al, (PMID: 10655498), Ito et al, (PMID: 11283351), Uetz et al, (PMID: 10688190), Ho et al, (PMID: 11805837), Gavin et al, (PMID: 11805826), Tong et al, (PMID: 14764870), Davierwala et al, (PMID: 16155567), Gavin et al, (PMID: 16429126), Schuldiner et al, (PMID: 16269340), Krogan et al, (PMID: 16554755), Pan et al, (PMID: 16487579) and Miller et al, (PMID: 16093310).

#### Reactome and orthology

Human protein-protein interaction pairs as defined by Reactome were downloaded from http://www.reactome.org/download/current/homo_sapiens.interactions.txt.gz on August 19 2006. According to the Reactome annotation standard, protein pairs in direct complex are not necessarily directly interacting but are part of the same core complex, while indirect complex means that two proteins are in the same meta-complex, i.e. two direct complexes that under certain cellular conditions associate (for example the TFII transcription factors and RNA polymerase II forming the pre-initiation complex). We extracted protein pairs which were designated ‘direct complex’ as interaction type and excluded protein pairs designated ‘indirect complex’. We only kept protein pairs assigned ‘direct interaction’, because we want only core complex proteins to keep our definition strict.

Orthology data was retrieved from the Ensembl database [25] version 41 using the BioMart mining tool (http://www.ensembl.org/biomart/martview/ accessed on October 26 2006). For deriving orthology Ensembl uses a pipeline for ortholog/paralog prediction based on best reciprocal similarity relationship as of June 2006. The method includes determining gene families by best reciprocal match, tree construction by PHYML and MUSCLE and tree reconciliation by the RAL algorithm. We have provided results based on orthology defined by the inparanoid program [18] in the supplementary material (Text S1). Although inparanoid is a less-advanced orthology inference method than Ensembl it shows slightly higher conservation of co-complex memberships.

In case of inparalogs in yeast interaction between the human protein pair was inferred from yeast when at least one combination of the yeast orthologs has an interaction according to the interaction dataset. We assumed that if one of the combinations has an interaction, the interaction is conserved in evolution and the other orthologs have lost the interaction after function divergence. This means that the conserved interaction does not have to be between orthologs which are closest in sequence which is consistent with Notebaart et al. [26] who state that an ortholog which has identical function, does not necessarily have to be closest in sequence.

### Data Handling

All data was handled by Perl scripts (Perl 5.8.8) on a 64 bit Linux machine.

## Supporting Information

Text S1 Supplementary Material.
(0.13 MB PDF)
