# Supplementary material for: Protein Complex Evolution Does Not Involve Extensive Network Rewiring
Source: PLoS Comput Biol. 2008 Jul 25;4(7):e1000132. doi: 10.1371/journal.pcbi.1000132 (PMC2517612; doi:10.1371/journal.pcbi.1000132)
Supplement: Text S1 — Supplementary Material. (0.13 MB PDF) [file pcbi.1000132.s001.pdf]

Determining optimal cut-off value for TAP-MS datasets

There is a tradeoff between the false negative rate and the false positive rate when determining the optimal cut-off value for the TAP-MS datasets. Because false positives are hard to determine due to the lack of larger reference sets, we have calculated a Receiver-Operator curve based on the agreement of two complex definitions, GO and MIPS. We have defined as positive interacting protein pairs two proteins in the same complex according to both MIPS and GO. Negative interacting protein pairs are defined as two proteins of which each is in another complex according to both MIPS and GO. As our benchmark dataset we have used the intersection dataset. We have also plotted Y2H datasets by Uetz et al. and Ito et al. for comparison to the Intersection dataset based on Krogan et al. and Gavin et al.. From this curve we find that a cutoff value of 0 is optimal for our question.

Table: Effect of cut-off on FN and FP

Cutoff	TP	FP	FN	TN
0	7130	33960	2481	185684
1	6542	14431	3069	205213
2	5223	3252	4388	216392
3	3510	696	6101	218948
4	2834	199	6777	219445
5	2397	97	7214	219547
6	1933	66	7678	219578
7	1614	48	7997	219596
8	1301	33	8310	219611
9	989	23	8622	219621
10	734	15	8877	219629
11	514	11	9097	219633
12	310	4	9301	219640
13	193	2	9418	219642
14	111	0	9500	219644
15	53	0	9558	219644

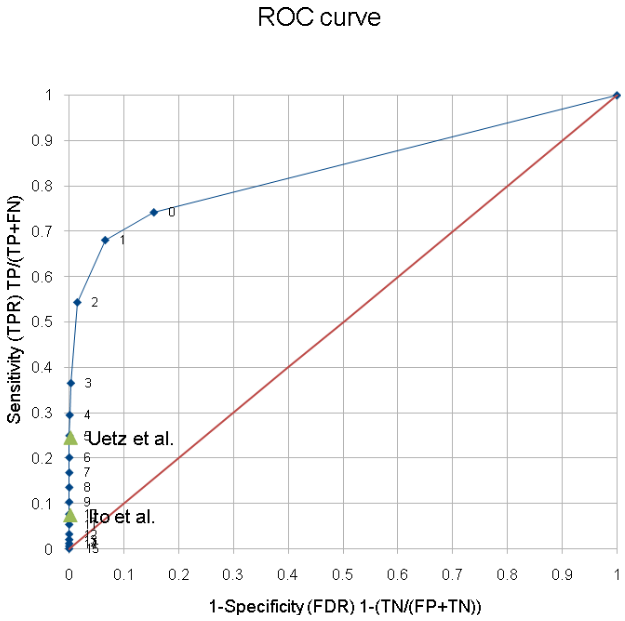

Ewing et al. HTP IP-HTMS dataset used to calculate conservation between human and yeast interactome

We have chosen Reactome as our reference set in human for calculating the conservation of co-complex membership because it is manually curated and based on expert opinion and therefore is likely to contain fewer errors. A new CoIP dataset for human by Ewing et al. has become available and we show here the same calculations when Reactome is substituted by this dataset below.

The authors state that interactions with a confidence score higher or equal to 0.3 should be regarded as high confidence. When using a higher cut-off value we see a steady rise in conservation (87% for >= 0.5 against the intersection dataset) but also see the total

number of conserved protein pairs plummet towards small numbers. The number of conserved protein pairs in Ewing when no cut-off value was used is significantly less than for Reactome and the conservation calculated is therefore less representative.

Ewing shows a much lower preservation of orthologs of protein pairs than Reactome (11% and 32% resp.). It is reported by Ewing et al. explicitly that they have based their bait selection on human disease association. Ewing therefore does not represent the basal conserved eukaryotic machinery as well as Reactome, which would account for the low conservation of protein pairs.

**Cut-off:** none	**Total interactions:**	5761	**Total conserved:**	650
**Dataset**	**PPI**	**NO-PPI**	**Conservation(%)**	**Coverage(%)**
Uetz	6	40	13.04	0.92
Ito	10	256	3.76	1.54
Uetz Int	6	3	66.67	0.92
Gavin	75	292	20.44	11.54
Krogan	154	450	25.50	23.69
Intersection	117	245	32.32	18.00
Inclusive	171	433	28.31	26.31

**Cut-off:** 0.3	**Total interactions:**	2039	**Total conserved:**	219
**Dataset**	**PPI**	**NO-PPI**	**Conservation(%)**	**Coverage(%)**
Uetz	5	10	33.33	2.28
Ito	9	63	12.50	4.11
Uetz Int	5	0	100.00	2.28
Gavin	58	81	41.73	26.48
Krogan	99	108	47.83	45.21
Intersection	78	59	56.93	35.62
Inclusive	105	102	50.72	47.95

**Cut-off:** 0.4	**Total interactions:**	695	**Total conserved:**	91
**Dataset**	**PPI**	**NO-PPI**	**Conservation(%)**	**Coverage(%)**
Uetz	2	3	40.00	2.20
Ito	5	22	18.52	5.49
Uetz Int	2	0	100.00	2.20
Gavin	33	18	64.71	36.26
Krogan	52	31	62.65	57.14
Intersection	37	14	72.55	40.66
Inclusive	53	30	63.86	58.24

**Cut-off:** 0.5	**Total interactions:**	245	**Total conserved:**	34
**Dataset**	**PPI**	**NO-PPI**	**Conservation(%)**	**Coverage(%)**
Uetz	0	1	0.00	0.00
Ito	2	5	28.57	5.88
Uetz Int	0	0	NA	0.00
Gavin	18	5	78.26	52.94
Krogan	26	5	83.87	76.47
Intersection	20	3	86.96	58.82
Inclusive	26	5	83.87	76.47

Orthology: results are not sensitive to orthology definition.

We also performed our analysis with another orthology definition. We have used inparanoid[1] to calculate orthology between human sequences from the UniProt database and yeast sequences from SGD. Inparanoid is a script which uses BLAST to obtain homology and calculated orthologs taking into account the existence of paralogs and in-paralogs. We have used the standard settings for inparanoid. Below is a table, like table 2 in the publication but based on the inparanoid orthology. We see that the orthology based on inparanoid results in slightly higher conservation and more conserved protein pairs. We feel that the orthology based on Ensembl is more advanced as it is based on reciprocal match, phylogenetic tree construction and tree reconciliation. We therefore used the Ensembl definition in our main analysis as opposed to InParanoid.

Conservation based on inparanoid (2596 conserved protein pairs)

Dataset	PPI	Non-PPI	Conservation	Coverage
Gavin	1646	274	85.73%	63.41%
Krogan	2084	462	81.85%	80.28%
Inclusive	2317	239	90.65%	89.25%
Intersection	1761	84	95.45%	67.84%
Uetz	23	83	21.70%	0.89%
Uetz Strict	23	4	85.19%	0.89%
Ito	37	634	5.51%	1.43%
**Human Dataset**	**PPI**	**Non-PPI**	**Overlap**	**Coverage**
Rual	2	9	18.18%	0.08%
Stelzl	5	111	4.31%	0.19%
Ewing	46	546	7.77%	1.77%

Conservation based on Ensembl (1916 conserved protein pairs)
Identical to table 2 in the main text

Dataset	PPI	Non-PPI	Conservation	Coverage
Gavin	1305	226	85.24%	68.11%
Krogan	1547	328	82.51%	80.74%
Inclusive	1717	167	91.14%	89.61%
Intersection	1392	75	94.89%	72.65%
Uetz	21	63	25.00%	1.10%
Uetz Strict	21	4	84.00%	1.10%
Ito	36	381	8.63%	1.88%
**Human Dataset**	**PPI**	**Non-PPI**	**Overlap**	**Coverage**
Rual	3	5	37.50%	0.16%
Stelzl	4	79	4.82%	0.21%
Corrected Ewing	30	420	6.67%	1.57%

Errors in orthology, complex definition and neo-functionalisation

Of the 167 non-interactions as found using Reactome and the Inclusive dataset, 139 appear to be potential false negatives. The remaining 28 non-conserved interactions consist of errors in orthology of one gene (5 interactions), incorrect assignment of two proteins to a complex in Reactome (10 interactions) and possible neo-functionalisation after duplication in human (3 proteins, 13 interactions).

Five protein pairs do not show an interaction due to incorrect orthology assignment in Ensembl. The human protein *TF2H4* [Swiss-Prot:Q92759] is annotated as orthologous to *VAS1* [SGD:YGR094W] and is present in five conserved protein pairs in Reactome. We could not confirm any homology between these proteins (let alone orthology) and it seems unlikely as well from the annotation: TF2H4 is a subunit of Transcription Factor IIH complex whereas *VAS1* is a valyl-tRNA synthetase.

Ten protein pairs are probably erroneously assigned to the spliceosome complex in Reactome, based on our re-analysis of the available literature. Of these ten pairs, five protein pairs contain *NFX1* [Swiss-Prot:Q12986] and five protein pairs contain *SMC1 alpha* [Swiss-Prot:Q14683]. Human *NFX1* [Swiss-Prot:Q12986] is associated with the spliceosome complex and “Export Receptor bound mature mRNA Complex” according to Reactome (internal id's 72022, 72074, 72057, 159329, 159259, 113815). *NFX1* however is a transcription factor for MHCII genes and is not implicated in pre-mRNA modifications or nuclear export. Confusingly the *NXF1* protein (mind the spelling of *N<u>XF</u>1*) is a known nuclear export factor. *NXF1* is not listed as part of the “Export Receptor bound mature mRNA Complex”. A miss spelling of *NXF1* might have caused a mix-up in Reactome. (for example *NXF1* [Swiss-Prot:Q9UBU9] is misspelled in Cohen and Panning[2] as *NFX1*.)

SMC1 alpha (human [Swiss-Prot:Q14683], yeast [SGD:YFL008W]), responsible for another five protein pairs, is part of the cohesin complex but also takes part in the spliceosome formation according to Reactome (internal id's 72159, 72022, 72074, 77505, 72057). However, we could not find any literature which linked *SMC1 alpha* directly to the spliceosome. The link between the cohesin complex and the spliceosome is one of its alleged co-complex members *CD2B2* [Swiss-Prot:O95400] of which *LIN1* [SGD:YHR156C is its ortholog in yeast. *LIN1* is implicated to link chromatine modification and the cohesin complex to the spliceosome complex[3]. But the similarity between *CD2B2* and *LIN1* is weak, and both have very different functions. *CD2B2* is involved in immunity and binds to antibodies, whereas *LIN1* is a non-essential component of U5 snRNP. *CD2B2* and the spliceosome are mentioned together in an article by Monos et al.[4] because an antibody raised against *CD2B2* also reacted with the spliceosomal *Sm B/B'* proteins. The experimental link between *SMC1 alpha* and the spliceosome is weak and it can therefore be argued that *SMC1* is not part of the spliceosome complex.

We identified 13 protein pairs which could be possible new interactions. Each of these

pairs contain one of three proteins: *PCBP1* [Swiss-Prot:Q15365], *PABP2* [Swiss-Prot:Q86U42] and *XAB2* [Swiss-Prot:Q9HCS7]. The human *PCBP1* is involved in regulating the spliceosome[5]. Its yeast ortholog *PBP2/HEK1* [SGD:YBR233W] is involved in the regulation of telomere position effect and telomere length[6]. However *PCBP1* is not the only ortholog of *PBP2*. 14 human proteins are orthologs to *PBP2*. These are active in different processes, some of them still perform the ancestral function[7]. So whereas *PBP2* solely has a function in the regulation of telomere position effect and telomere length in yeast, the human *PCBP* family of inparalogs has gained many other functions and interaction partners after several rounds of duplications in the course of evolution (neofunctionalization of inparalogs).

The human *PABP2* is a poly(A)-binding protein and is part of the “3' end cleaved, ligated exon containing complex” in the nucleus according to Reactome. Its ortholog in yeast, *SGN1* [SGD:YIR001C], is a poorly characterized poly(A)-binding protein that localizes to the cytoplasm and not to the nucleus[8]. Hence some degree of functional differentiation took place in either human or yeast.

The human protein *XAB2* is involved in transcription coupled-nucleotide excision repair (TC-NER)[9] and also in mRNA splicing (spliceosome)[10] albeit indirectly. The ortholog of *XAB2* in yeast, *SYF1* [SGD:YDR416W], is a component of the spliceosome[11, 12]. *SYF1* however has not been implied with nucleotide excision repair. *XAB2* apparently has gained a new function, and new interaction partners, in human TC-NER, but also seemingly retained its ancestral function (or some of it), like its yeast ortholog *SYF1*, in the spliceosome.

References

1. **Remm, M., C.E. Storm, and E.L. Sonnhammer, *Automatic clustering of orthologs and in-paralogs from pairwise species comparisons.* Journal of Molecular Biology, 2001. 314(5): p. 1041-52.**

2. **Cohen, H.R. and B. Panning, *XIST RNA exhibits nuclear retention and exhibits reduced association with the export factor TAP/NXF1.* Chromosoma, 2007. 116(4): p. 373-83.**

3. **Bialkowska, A. and A. Kurlandzka, *Proteins interacting with Lin 1p, a putative link between chromosome segregation, mRNA splicing and DNA replication in Saccharomyces cerevisiae.* Yeast, 2002. 19(15): p. 1323-33.**

4. **Monos, D., et al., *Analysis of the CD2 and spliceosomal Sm B/B' polyproline-arginine motifs defined by a monoclonal antibody using a phage-displayed random peptide library.* J Mol Recognit, 2006. 19(6): p. 535-41.**

5. **Meng, Q., et al., *Signaling-dependent and coordinated regulation of transcription, splicing, and translation resides in a single coregulator, PCBP1.***

Proc Natl Acad Sci U S A, 2007. 104(14): p. 5866-71.

6. **Denisenko, O. and K. Bomsztyk, *Yeast hnRNP K-like genes are involved in regulation of the telomeric position effect and telomere length.* Mol Cell Biol, 2002. 22(1): p. 286-97.**

7. **Makeyev, A.V. and S.A. Liebhaber, *The poly(C)-binding proteins: a multiplicity of functions and a search for mechanisms.* Rna, 2002. 8(3): p. 265-78.**

8. **Winstall, E., et al., *The Saccharomyces cerevisiae RNA-binding protein Rbp29 functions in cytoplasmic mRNA metabolism.* J Biol Chem, 2000. 275(29): p. 21817-26.**

9. **Nakatsu, Y., et al., *XAB2, a novel tetratricopeptide repeat protein involved in transcription-coupled DNA repair and transcription.* J Biol Chem, 2000. 275(45): p. 34931-7.**

10. **Yonemasu, R., et al., *Disruption of mouse XAB2 gene involved in pre-mRNA splicing, transcription and transcription-coupled DNA repair results in preimplantation lethality.* DNA Repair (Amst), 2005. 4(4): p. 479-91.**

11. **Ben-Yehuda, S., et al., *Genetic and physical interactions between factors involved in both cell cycle progression and pre-mRNA splicing in Saccharomyces cerevisiae.* Genetics, 2000. 156(4): p. 1503-17.**

12. **Russell, C.S., et al., *Functional analyses of interacting factors involved in both pre-mRNA splicing and cell cycle progression in Saccharomyces cerevisiae.* Rna, 2000. 6(11): p. 1565-72.**
